# Scaling depth from shadow offset

**DOI:** 10.1167/jov.21.12.15

**Published:** 2021-11-29

**Authors:** Patrick Cavanagh, Roberto Casati, James H. Elder

**Affiliations:** 1 Centre for Vision Research, York University, Toronto, ON, Canada; 2 Department of Psychology & Department of Electrical Engineering and Computer Science, York University, Toronto, ON, Canada; 3 Institut Jean Nicod, Département d'études cognitives, ENS, EHESS, CNRS, PSL University, Paris, France; 4 Department of Psychology, Glendon College, Toronto, ON M4N3M6, Canada; 5 Department of Psychological and Brain Sciences, Dartmouth College, Hanover, NJ 03755, USA

**Keywords:** depth, shadows

## Abstract

When an object casts a shadow on a background surface, both the offset of the shadow and the blur of its penumbra are potential cues to the distance between the object and the background. However, the shadow offset and blur are also affected by the direction and angular extent of the light source and these are often unknown. This means that the observer must make some assumptions about the illumination, the expected distribution of depth, or the relation between offset and depth in order to use shadows to make distance judgments. Here, we measure human judgments of perceived depth over a range of shadow offsets, blurs, and lighting directions to gain insight into this internal model. We find that distance judgments are relatively unaffected by blur or light direction, whereas the shadow offset has a strong and linear effect. The data are consistent with two models, a generic shadow-to-depth model and a Bayesian model.

## Introduction

**Figure 1.**
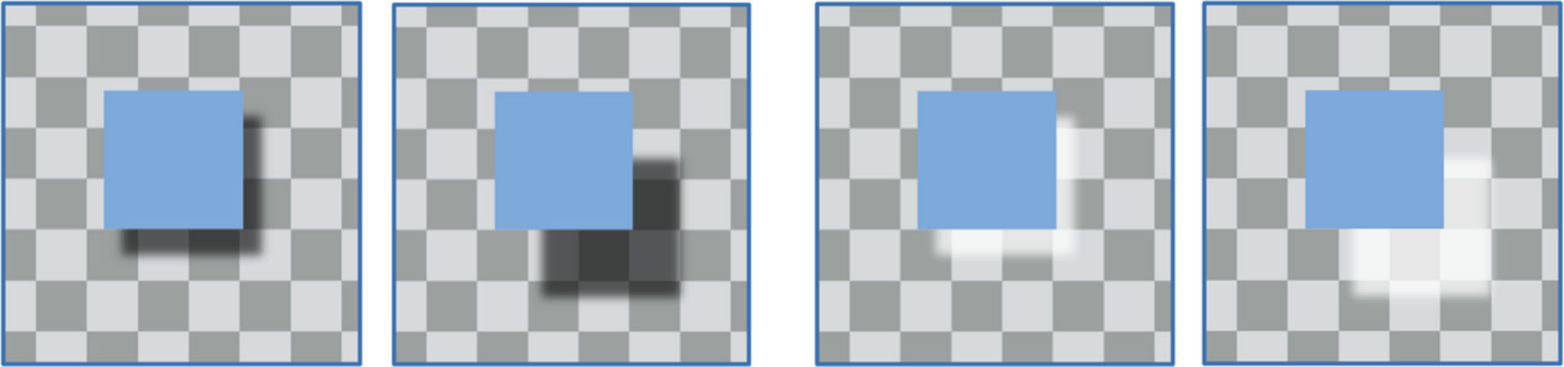
Left. When the L-shaped region adjacent to the blue square is darker than its surround, it is taken as a shadow and creates the impression of a separation between the square and the checkered background. This perceived separation increases when the dark patch is shifted further from the blue square. Although this shift in shadow position could be caused by either a change in the position of the light source or a change in the height of the blue square above the background, perception strongly favors the latter account (Kersten et al., 1996). Right. Note that if the same L-shaped region is lighter than its surround, there is no impression of a shadow and little or none of a separation between the square and the background. In other words, the impression of depth is specific to the processing of valid shadows.

The position of a cast shadow depends on the direction of illumination and the separation of the object from the surface on which its shadow falls. If any two of these three parameters are known (shadow position, illumination direction, and separation of object from background), the third can be recovered. However, it is often the case that the direction of illumination is unknown, making the estimation of separation between the object and the shadowed surface underdetermined. This does not stop human observers in such situations – we experience a clear separation that is relatively easy to quantify (see Figure 1) and we will examine these depth reports in the experiment here. A previous study by Kersten, Knill, Mamassian, and Bülthoff (1996) has already shown that changes in shadow offset generate an impression of movement of the object casting the shadow. This result suggests that the human visual system makes some assumptions about the direction of the illumination or the link between shadow offset and depth, but what assumptions? In the two left hand panels of Figure 1, we have a sense that the light is above us and to our left – it could hardly be elsewhere for the shadow to be below and to the right of the blue square. But our visual system must be performing a more quantitative analysis to explain the clear difference in depths seen in Figure 1 (left). How can we best understand this analysis?

One approach is that the visual system assumes a generic, single light source direction, the same for every scene or image, and then uses this together with the shadow offset to recover the separation of the object from its background — a generic shadow-depth (GSD) model. In this case, the recovered depth would always scale linearly with the shadow's offset. The proposal of a single light source is controversial (Gilchrist, 2018) because there are often multiple light sources in a scene and there are many examples of the perception of depth from shadows where the shadows are not consistent with any light source (e.g. Ostrovsky, Cavanagh, & Sinha, 2005; Mamassian, 2004). To address this, we will consider two versions of this GSD model, one with and one without an assumption of a light source. The outcome for both is the same: depth scales linearly with shadow offset. The advantage of the version without the light source is that it allows multiple, inconsistent shadows to each generate their own depth estimates so that it easily accommodates inconsistency across shadows.

A second approach is to find the separation between the object and its background that is most consistent with the shadow offset across the range of possible illumination directions, weighting the likelihood of different separations by their expected distribution in natural scenes. We will evaluate the predictions of this Bayesian model along with those of the GSD model in the discussion.

**Figure 2.**
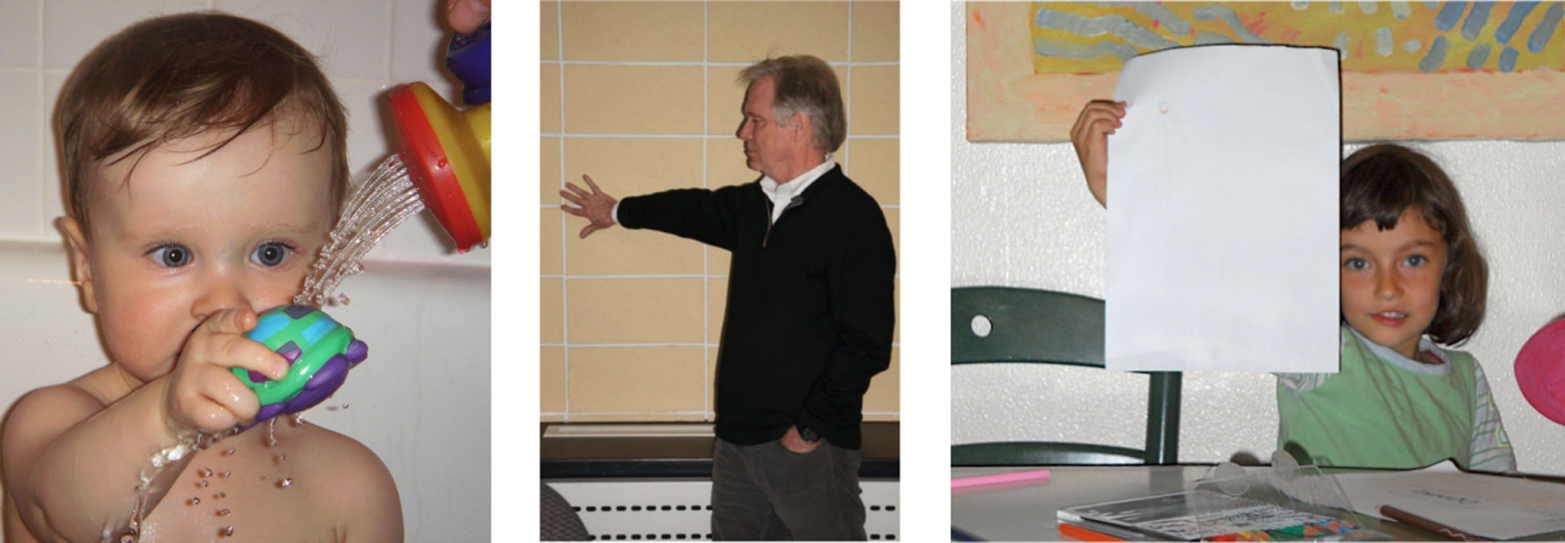
Flash photographs generate very tight shadows due to the small offset between the camera lens and the flash, leading to greatly compressed perceived depth. On the left, the shadows of the water droplets falling from the toy appear very close to the toddler's chest when in fact they are at arm's length, about 30 cm. The shower head appears close to the wall behind but must be near the extended hand. In the middle, the narrow shadow to the left of the man's head makes him appear to be very close to the wall and, as a result, his arm appears to stick out straight from his body, rather than stretching 50 cm to his right as it actually does. This makes his hand appear abnormally small (the Trump illusion). On the right, the tight shadow makes the paper appear impossibly close to the background even though the girl is holding it out at arm's length in front of her, 1.5 m from the wall.

Whether the brain infers a specific shadow-to-depth relation or estimates depth across a range of possible light directions, it seems to produce reasonable depth estimates in scenes where the light source and the object distributions are within the “normal” range. However, in a scene that has a light source at an unusual position, the underlying assumptions lead to misjudgments of depth. Flash photography, for example, typically produces “tight shadows” because the light source is near the camera lens (Figure 2). These tight shadows make the separation between the object and the background appear much smaller than it is (see Figure 2). These odd impressions of compressed depth in photographs suggest that the visual system does not recover the correct light source direction from these images but relies instead on some assumptions about the scene or the relation between shadow offset and depth.

**Figure 3.**
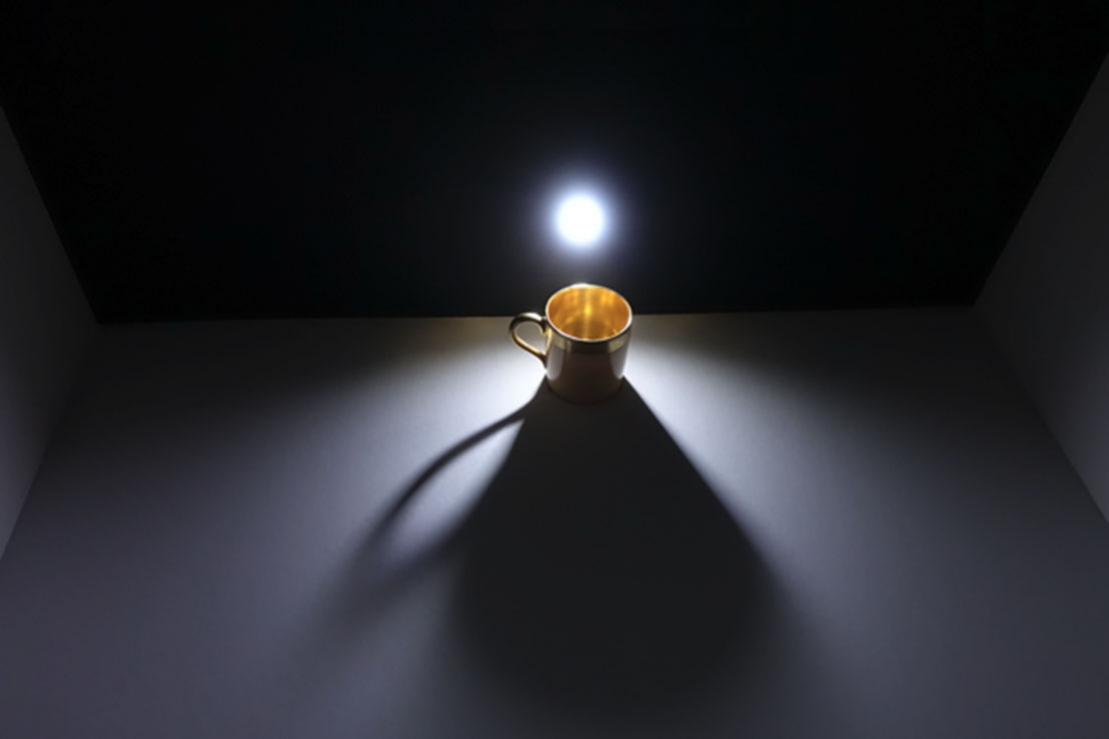
The penumbra at the shadow's edge widens as the distance increases between the cup and the surface on which the shadow cast by the cup falls.

Shadow offset is only one of the shadow properties that might influence the perception of depth. Another is the sharpness of the shadow boundary. Natural shadows have boundaries with a gradient from dark to light (Figure 3). The visual extent of this gradient is determined by many factors: the angular extent of the light source, the distance between the object and the background, and the distance and orientation of the background surface relative to the eye. The border can be sharp if an object casts a shadow on a nearby surface or if the illumination is a point source. Alternatively, the border can be extremely broad with a very shallow luminance gradient if the shadow falls on a distant surface or the light source is diffuse. Nevertheless, when other factors are fixed there is a direct relation between the blurriness of a shadow border and the distance between the object and the surface on which the shadow is cast. The greater the distance to the shadowed surface, the blurrier the shadow edge. In addition to demonstrating that perceived depth increases with the offset of a moving shadow, Kersten et al. (1996) and Mamassian, Knill, and Kersten (1998) showed that blurry shadows are more effective at triggering an impression of motion in depth. Here, we will ask whether humans are able to use this cue quantitatively in a static stimulus – whether there is any scaling of apparent depth as the blur of the shadow border is increased.

Finally, the effect of the shadow on perceived depth may depend not only on the offset and blur of the shadow but also on its direction relative to the object, reflecting the well-established biases that favor lighting from above (Ramachandran, 1988; Adams, Graf, & Ernst, 2004; Jenkin, Jenkin, Dyde, & Harris, 2004; Adams, 2007; Stone, 2011) and perhaps to the left (Sun & Perona, 1998; Mamassian & Goutcher, 2001). However, these studies dealt mostly with the interpretation of ambiguous shaded surfaces, not cast shadows. Kersten et al. (1996) did test cast shadows and they too reported that light from above was more effective at generating an impression of motion in depth compared to light from below. However, we do not yet know whether the direction of the shadow also affects the perception of depth in static stimuli.

## Method

### Participants

Twenty-five healthy adults took part in the experiment (4 men and 21 women, mean age = 22 years, SD = 2.1, with a range of 19 to 38). All participants were undergraduates at Glendon College and naïve to the purpose of the experiment. All participants reported normal or corrected-to-normal vision and all gave informed consent in writing prior to participation. The protocols for the study were approved by the York University Review Board in accordance with the principles of the Declaration of Helsinki (2003).

### Stimuli and apparatus

**Figure 4.**
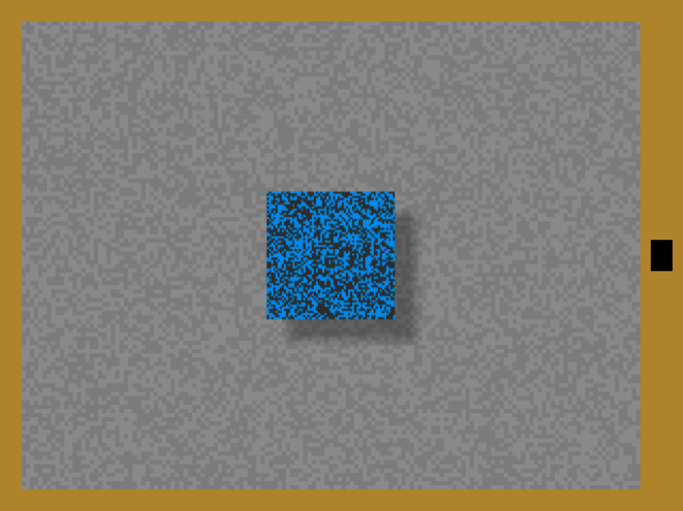
The blue, textured square had a shadow with one of six offsets, one of three blurs, and one of four directions (here 0.38 dva offset, bottom right, 0.25 dva of blur). Participants adjusted the height of the black bar on the right to match their impression of the depth separating the square from the background.

The experiment took place in a darkened room. Stimuli were presented on a gamma-corrected CRT monitor (85 Hz, 800 × 600 pixel resolution covering 26.6 × 20 cm) controlled by a Macintosh G4 laptop running Vision Shell Graphics libraries (Comtois, 2003). Participants were seated 1 m from the monitor with their heads resting on a chin- and headrest and viewed the stimuli binocularly. A background of low contrast, grey, random noise covered 14.15 degrees of visual angle (dva) × 10.75 dva with the noise elements being 0.04 dva square. A 2.30 dva square was centered in the display (Figure 4). The square was filled with high contrast black and blue random noise. The noise elements were 0.08 dva square. Shadows were displaced diagonally from the square in the four diagonal directions, with one of six offsets from 0.14 dva to 2.16 dva. Shadows took one of three blur levels, generated by convolving the shadow with a blur circle of 1, 13, or 27 pixels diameter (0.02 dva, 0.25 dva, or 0.52 dva) to simulate the effect of spherical light sources of different spatial extents. The blur circle of one pixel simulated a point source, producing a sharp shadow. The shadows were rendered as a 50% reduction of luminance of each background pixel – tapering to 0% reduction at the edge of the blur. There was also a no-shadow control. The mean luminance of the grey background was 62.3 cd/m^2^ and that of the blue (cyan) square was 36 cd/m^2^.

### Procedure and design

**Figure 5.**
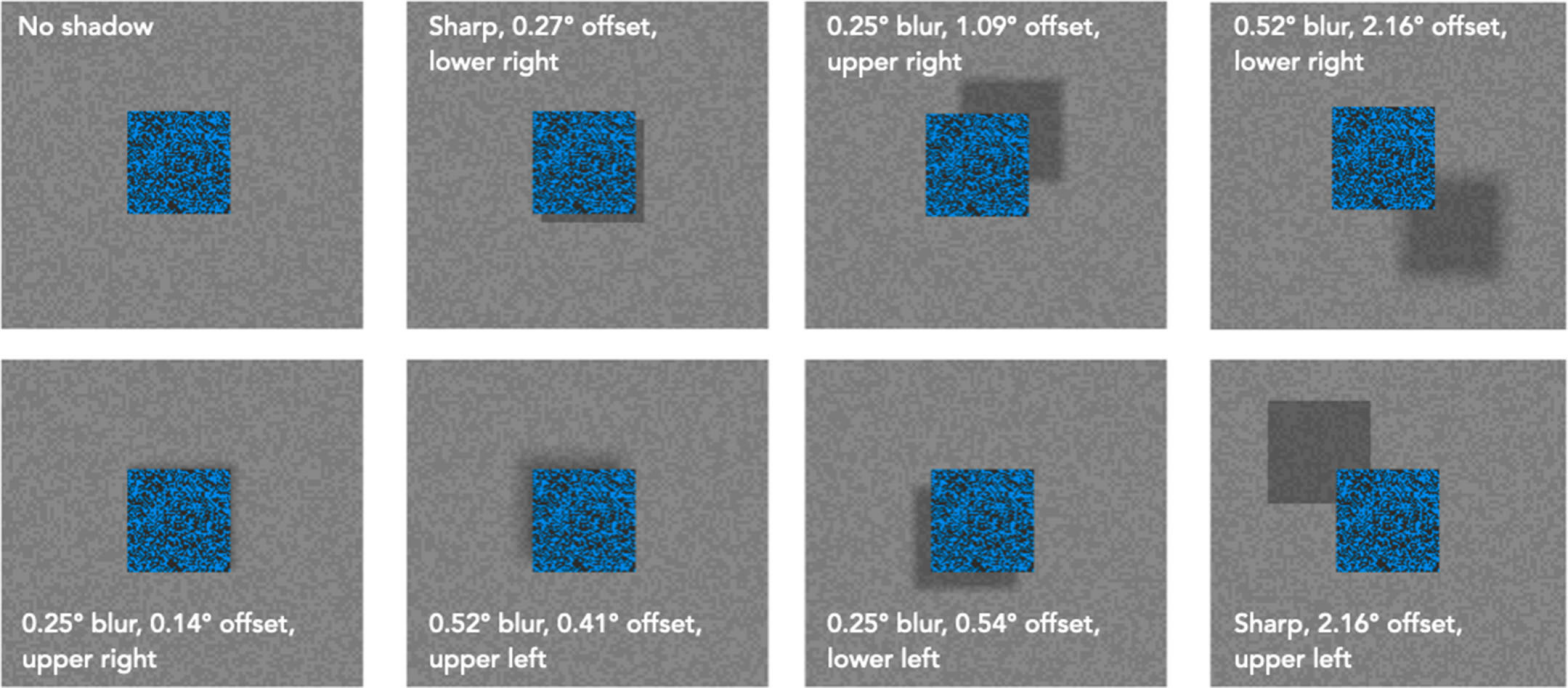
Sample stimuli. On each trial the grey background texture was different but the blue square's texture remained the same.

Participants performed 300 method-of-adjustment trials, four trials in each of the 72 conditions (6 offsets, 4 directions, and 3 blurs) and 12 control trials with no shadow (see Figure 5). During each trial, the square appeared in the center of the display with its shadow. Participants were instructed to first decide if they saw any depth separation between the square and the background. If they did, they were instructed to imagine sliding a tablet of paper under the square and estimate how thick the tablet could be to just fit. They then adjusted the height of the marker on the right of the display to match their estimate – or set it to zero height if they saw no depth. There was no time limit and participants were free to move their eyes wherever they wanted. When the participant was satisfied with the setting, he or she pressed the space bar to end the trial. The duration of each trial depended on the participant but averaged about 5 seconds. The order of the conditions across trials was random for each participant. The session lasted approximately 20 minutes.

## Results

**Figure 6.**
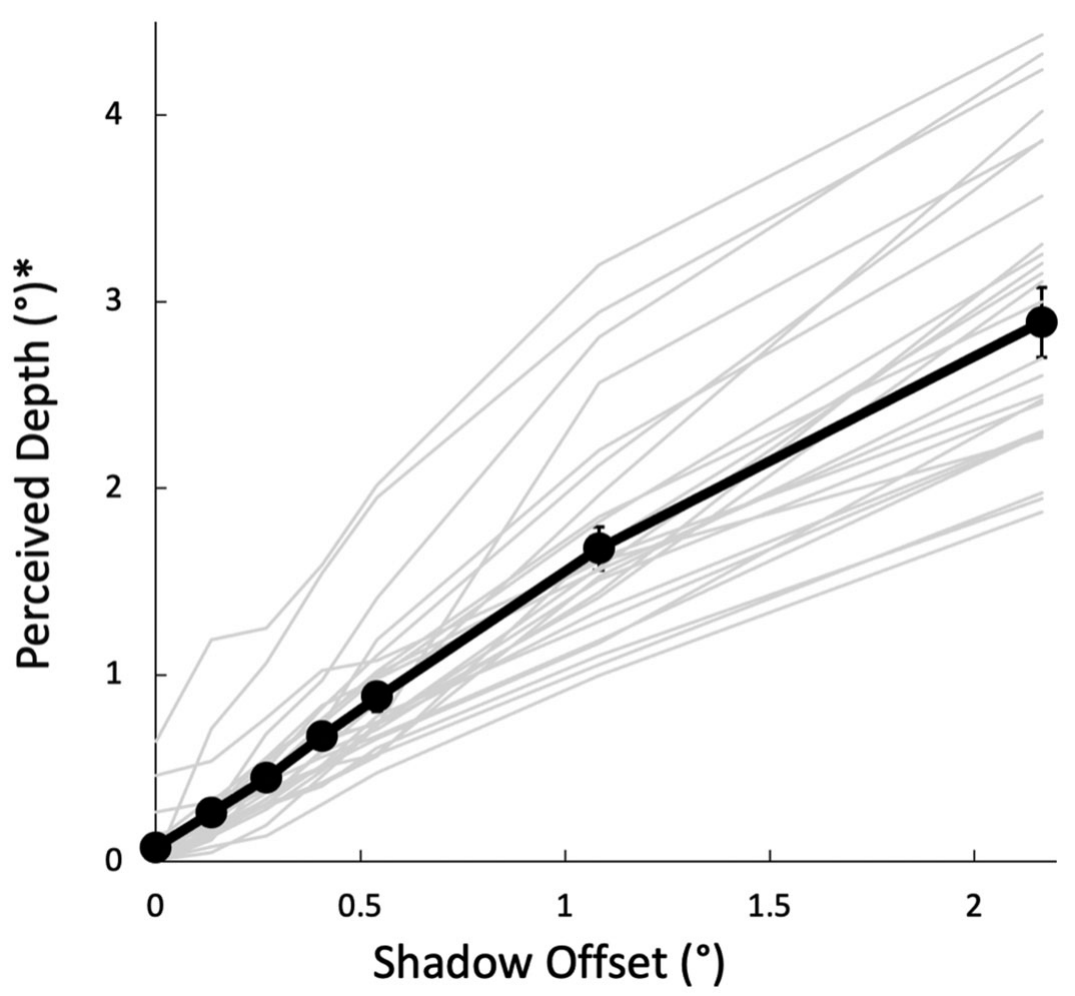
Perceived depth as a function of shadow offset, averaged across blur and offset direction. Perceived depth is, on average, about 30% greater than the shadow offset (slope of the linear fit to group data = 1.31). Average over 25 participants shown in black with ±1 SE bars. Individual participants shown in gray. * The perceived depth taken from the marker settings is given in degrees of visual angle of the marker in the frontoparallel plane.

**Figure 7.**
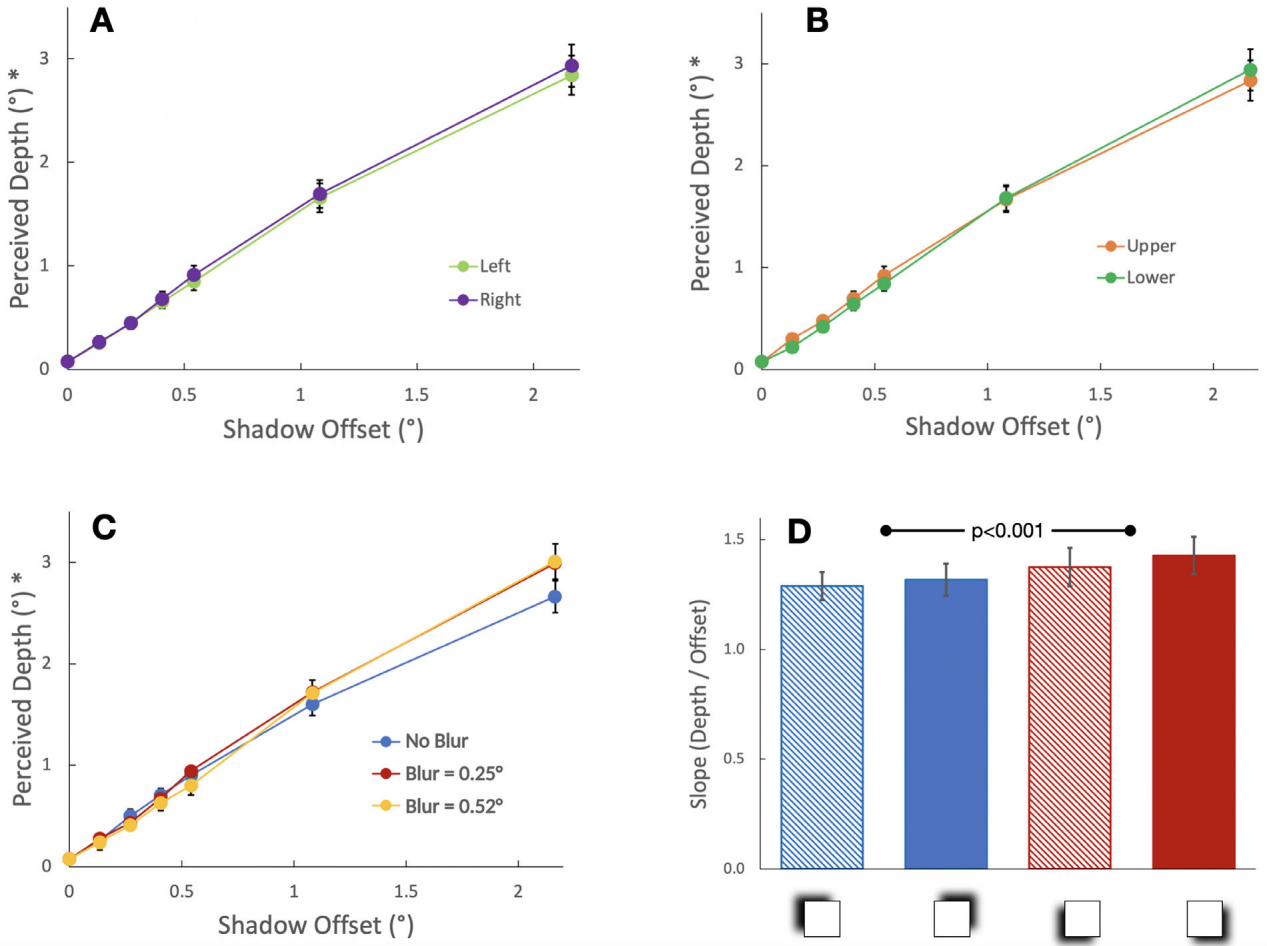
(**A**) Perceived depth as a function of shadow offset averaged over the three blur conditions, showing left and right offsets separately with ±1 SE bars. The plots here and in panels **B** and **C** repeat the same control value for shadow offset of zero, although these values were not included in the ANOVA. (**B**) Upper and lower offsets plotted separately, averaged over the three blur conditions. (**C**) The three levels of blur plotted separately, averaged over the 4 directions of offset. (**D**) The slopes of the regressions fit to the six offsets, excluding the no shadow control, individually for each participant. The data are averaged over the three blur conditions. The horizontal bar indicates that the average slopes for shadows below (light from above) was significantly higher than for the average for shadows above (light from below).

A 3-way ANOVA was run on the depth estimates with four levels of direction, three levels of blur and six values of offset (the 0 offset control was not included in this analysis because neither blur nor direction are defined when there is no shadow). The depth estimates for the 25 participants showed a strong (*F*(5,120) = 280.2, *p* < 0.001, *η_p_^2^* = 0.92, *P_r_* = 1.0) increase with the offset of the shadow (Figure 6). The effect of offset accounted for the major part of the data variance (*η^2^* = 0.57). Only three other effects reached significance — left versus right shadow offset, the interaction of offset with upper versus lower shadow position, and the interaction of offset with blur — and these explained far less of the variance (*η^2^* of 0.0002, 0.0035, and 0.0008, respectively). Specifically, there was a small but significant increase in depth (1.6%) for shadows on the right (equivalent to light from the left) versus on the left (Figure 7A; *F*(1,24) = 10.6, *p* = 0.003, *η_p_^2^* = 0.31, *P_r_* = 0.88). Second, there was a small but significant interaction between offset size and upper versus lower shadows (*F*(5,120) = 5.1, *p* < 0.001, *η_p_^2^* = 0.18, *P_r_* = 0.98) with the lower shadows (equivalent to light from above) producing a slightly steeper slope (Figure 7B). Finally, there was an interaction between blur and offset size (*F*(10,240) = 4.6, *p* < 0.001, *η_p_^2^* = 0.16, *P_r_* = 0.99) with the no-blur results having a slightly lower slope than the two blurred cases (Figure 7C). No other main effects or interactions were significant.

The control condition with no shadow was frequently judged as having no depth — 54% of all responses were zero. The rest of the settings indicated that some depth was seen by some observers on some trials. The average depth setting across all participants and trials was 0.07 ± 0.03 dva significantly greater than zero (*t*(23) = 2.34, *p* = 0.014) although the validity of the test is questionable as the distribution of settings is far from normal (the settings, perceived depth, cannot be negative).

We also analyzed the linear slope of depth versus offset. The slope fit to the average depth estimates across participants and conditions (including the 0 offset control condition) shown in Figure 6 is 1.31 ± 0.04 and the intercept was 0.12 ± 0.04 dva. When the slope was fit with an intercept of zero, it was 1.40 ± 0.05, consistent with a direction of illumination (slant) of 35.5 degrees away from the surface normal. We then analyzed the slopes separately for each condition and participant based on the six offsets, excluding the no-shadow control. Here, the average of slopes fit individually for each participant and condition was 1.35 ± 0.07 (Figure 7D) and ranged from 0.88 to 2.01. The difference in slopes for the four directions of shadow offsets is shown in Figure 7D. The slope for the no blur conditions (not shown) was 7.9% lower than the average for the two blurred conditions.

## Discussion

The results showed that perceived depth scaled quite linearly with shadow offset and was largely unaffected by the degree of blur or by the direction of the offset. Overall, the perceived depth was 30 to 40% larger than the shadow offset. This ratio of depth from offset varied considerably across participants, from 0.88 to 2.01, so it would be an overgeneralization to say that there is a bias for specifically 40% more depth than the offset. Nevertheless, the depth from offset relation is roughly linear not just in the aggregate, but for each individual participant (see Figure 6), and the mean ratio was significantly greater than 1 (*t*(24) = 4.71, *p* = 8.6 × 10^−5^). This relation between depth and offset is equivalent to a lighting direction of about 35.5 degrees from the head (GLD model, see details below), with a range of 26 degrees to 49 degrees across the participants.

Interestingly, there was little effect of the direction of the shadow offset. Although this seems at odds with previous reports of a bias of light from above (Ramachandran, 1988; Adams, et al., 2004; Jenkin, et al., 2004; Adams, 2007; Stone, 2011) and perhaps to the left (Sun & Perona, 1998; Mamassian & Goutcher, 2001), it has been argued that this bias is weak (Morgenstern, Murray, & Harris, 2011). In addition, these earlier studies mostly looked at convex/concave ambiguities from shading. In contrast, here, the sign of depth is clear: the square sits in front of the background.

In addition, we found only a small effect of shadow blur on perceived depth. These modest effects of shadow direction and blur are in contrast to the results of Kersten et al. (1996), who did find that a moving shadow elicited the perception of motion-in-depth more frequently when shadows were blurred and consistent with light from above. However, Kersten et al. did not measure the amplitude of the perceived motion. It is possible that the position and blur of the shadow have a greater effect on whether depth is perceived at all, rather than the magnitude of the perceived depth.

### Modeling

**Figure 8.**
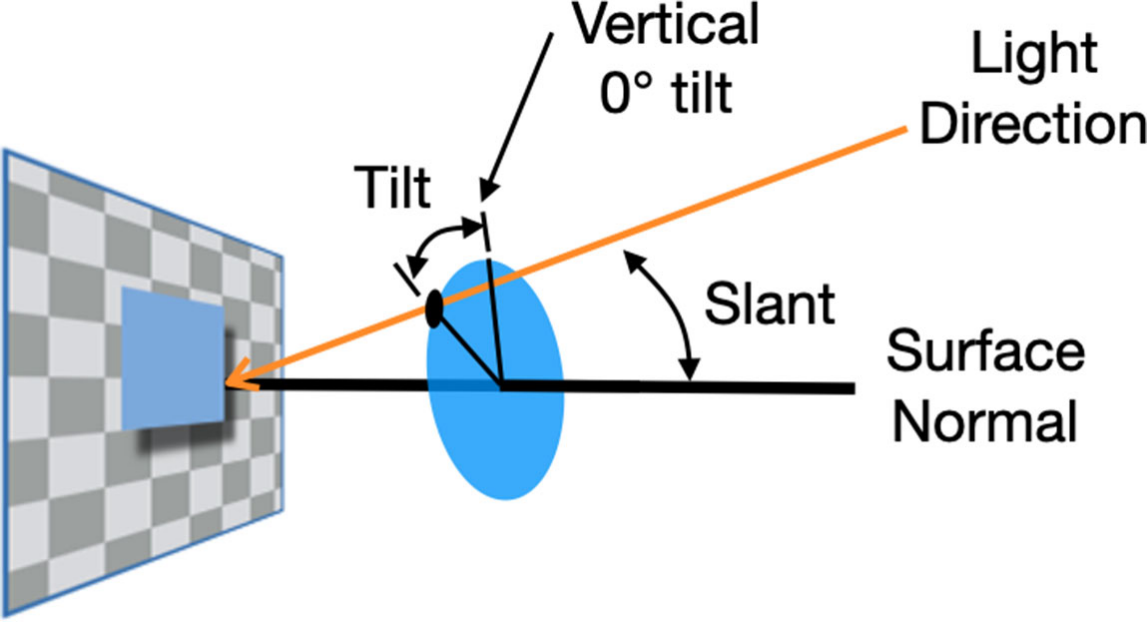
Slant and tilt of the light direction relative to the surface normal.

Our stimulus was a flat, vertical textured surface with a square lying on or in front of it, viewed directly so that the line of sight to the shadow was approximately aligned with the normal to the display surface. We characterize possible light directions in terms of the slant of the light source vector away from the surface normal and its tilt (i.e. the rotation of the light source vector around the surface normal; Figure 8). Although it may seem overkill to model data that are basically a straight line, we will do so and we have two proposals.

#### Generic shadow-to-Depth 

**Figure 9.**
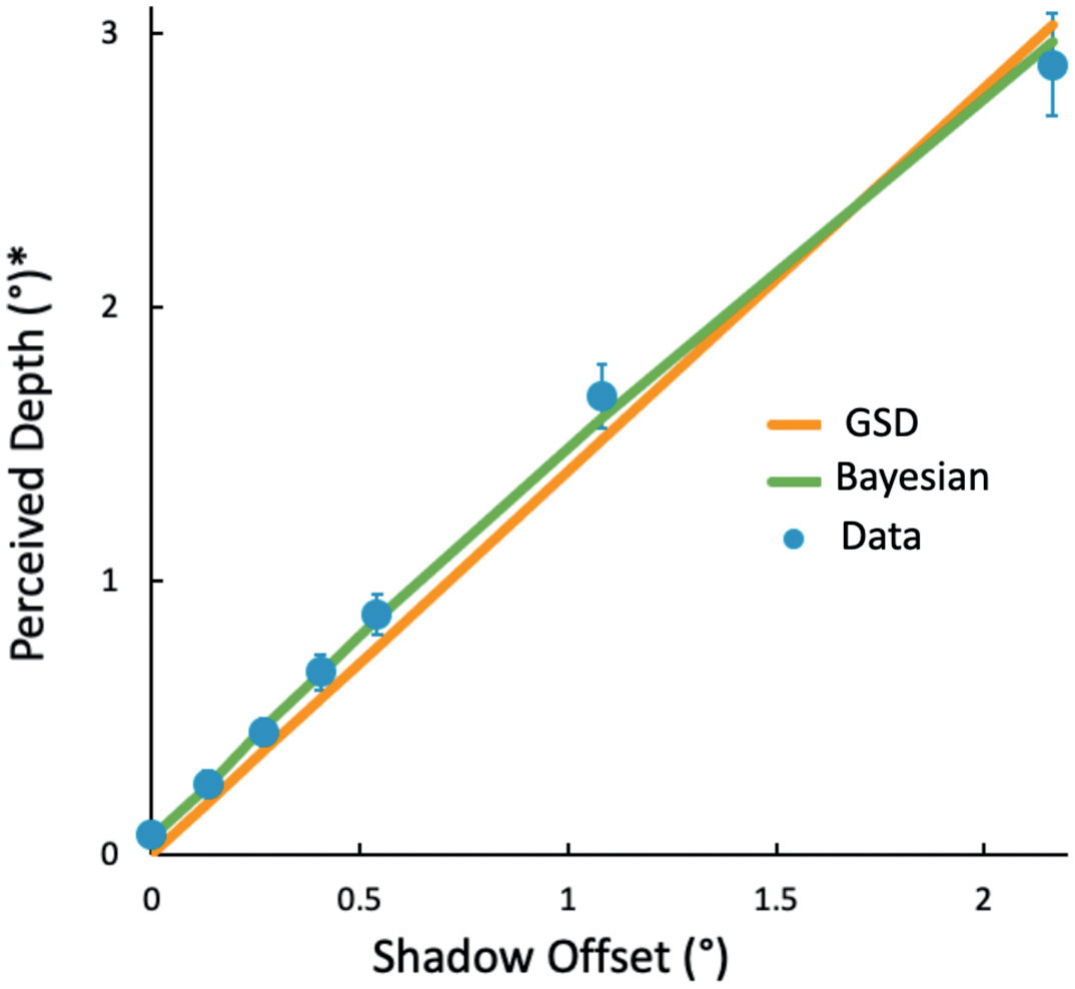
Fit of the generic shadow-depth (GSD) and Bayesian model to the data. Vertical bars are ±1.0 SE.

As mentioned in the Introduction, we consider two versions of this model, one with a light source assumption and one without. In the first case, in line with several authors (Ramachandran, 1988; Sun & Perona, 1998; Mamassian & Goutcher, 2001; Adams, et al., 2004; Jenkin, et al., 2004; Boyaci, Doerschner, & Maloney, 2006; Adams, 2007; Brainard & Maloney, 2011; Stone, 2011), we consider that the visual system recovers depth from shadows by assuming a single light source. In our stimuli, there is an unambiguous shadow shifted in an oblique direction. Any light source creating the shadow must therefore have a tilt around the surface normal that is opposite to the direction of the shadow offset. Although the tilt is set by the direction of the shadow's offset, the slant (angle between the light direction and the surface normal) is not. Here, we propose that the visual system assumes a specific, generic slant that is the same for all the four directions of offset and tilt. Because the slant is the same for all six offsets that we used for each direction, the shadow offset will be a fixed proportion of the separation between the object and the background: the greater the offset, the greater the separation. Depth then is linearly related to the shadow offset, dropping to no depth at zero offset. The GSD model has one free parameter: the slope of the depth versus offset relation with the intercept set to zero. The slope is 1.4, equivalent to a generic light source at 37.9 degrees of slant away from the surface normal. The fit is good (the orange line, GSD; Figure 9) with a root mean squared error (RMSE) of 0.11 degrees.

**Figure 10.**
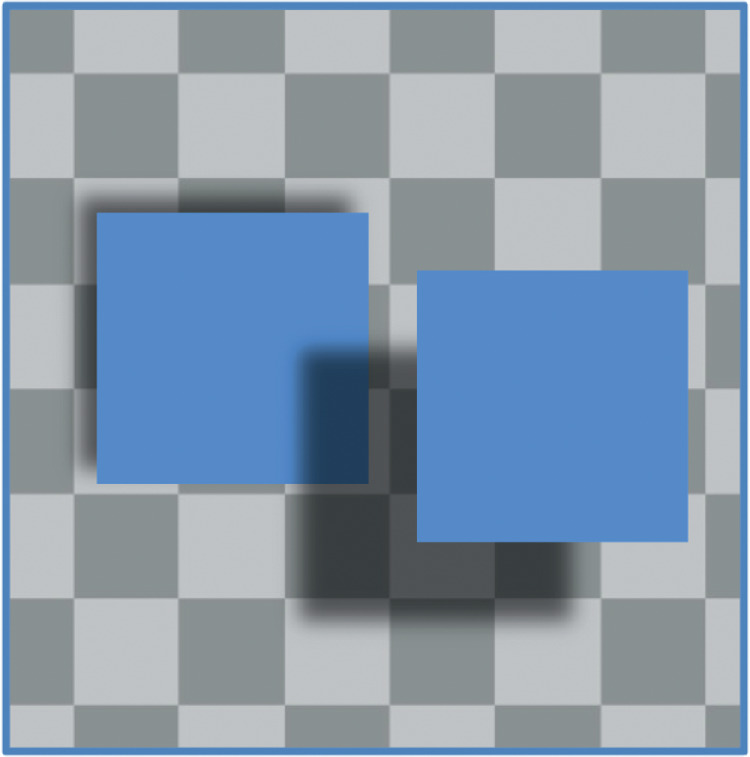
Two squares cast shadows in impossibly different directions. The left square is illuminated from the bottom right and the right square from the top right. Nevertheless, the two shadow offsets support corresponding depth impressions: the square on the right appears farther above the background than the square on the left, in line with the size of their shadow offsets.

Gilchrist (2018) and others have noted that the assumption of a single light source is problematic. In particular, there are many examples showing normal recovery of depth from shadows even when they are not consistent with any light source (Mamassian, 2004; Ostrovsky et al., 2005; Cavanagh, 2005; Casati & Cavanagh, 2019). Figure 10, for example, shows two inconsistent cast shadows that nevertheless support clear impressions of depth. The rightmost square appears farther from the background than the leftmost one, in agreement with their shadow offsets but inconsistent with any possible lighting, let alone any single light source. Given that the data show a linear scaling of depth with offset, a more parsimonious version of this model would bypass the physical model involving a light source. Instead, the visual system would learn to compute the depth directly as about 1.4 times the shadow offset — perhaps because that is the average in the real world. For this to be the case, the average direction of illumination in natural scenes would have to be about 35.5 degrees. The two versions of this model, with and without an explicit light source, have the same linear prediction but the light-free version has the advantage of being agnostic about inconsistent or impossible light sources (Figure 10).

#### Bayesian estimation

Given an observed shadow offset, the observer may be uncertain about the illumination direction and the corresponding depth. Any estimate of depth is unlikely to be exactly correct, but the visual system can still deliver an estimate that is optimal in minimizing the expected squared error without assuming or computing a specific light source direction. To do so, the visual system may compute the posterior distribution over the variable of interest (depth) conditioned on the observed variable (shadow offset). The posterior probability of a given depth estimate is proportional to the product of the likelihood of the shadow offset given the depth, and the prior probability of that depth. The uncertainty encoded by the likelihood function derives from two sources: noise in the estimated shadow offset, and uncertainty about the illumination slant. In the supplementary materials, we argue that the latter uncertainty dominates, so that the likelihood is determined by the prior over the illumination slant. The Bayesian model thus depends on the priors over both relief and illumination slant. For relief, we consider a flat prior and an empirical prior favoring small reliefs derived from the Southampton-York Natural Scenes (SYNS) dataset (Adams, Elder, Graf, Leyland, Lugtigheid, & Muryy, 2016; Ehinger, Adams, Graf, & Elder, 2017). For illumination slant, we consider a flat prior and a two-parameter beta distribution model that we fit to the human depth judgments. The Bayesian model estimates depth from shadows based on the whole range of possible light sources without estimating a specific light source direction. And why would it? Unless we are shielding our eyes from something bright, our actions seldom require a knowledge of the light source location.

We find that both the empirical prior over depth and the non-uniform illumination prior are required to fit the data as well as the GSD model (Figure 9). The estimated illumination prior is broad but not uniform, favoring moderate slants over very small slants (illumination coming from the direction of the observer) or very large slants (illumination grazing the visible surfaces), with a mean slant of 37.4 degrees. The other versions of the Bayesian model performed less well and are described in the supplementary materials.

Although both models provide a good quantitative account of mean human judgments, we note that there was considerable variation among participants, with slopes of perceived depth versus shadow offset ranging from 0.88 to 2.01. Future work could examine whether these large individual differences can be related to differences in lived experiences or related to other perceptual measures.

## Conclusions

Our results suggest that the perceived depth derived from a shadow is roughly a fixed proportion of its offset, at least for the small shadow offsets tested here. We also found little or no effect of different levels of blur or directions of the shadows. Both the GSD and Bayesian models fit the data closely and indicated an equivalent light direction of 35.5 degrees slant or a broad light prior with a mean of 37.4 degrees slant, respectively. There was no statistical difference between the predictions of the two models. Our findings make it clear why flash photographs (see Figure 2) create such a flattening of depth — with many cameras, the flash is near the camera lens, producing very tight shadows, which in turn yield a percept of shallow depth. We would expect the opposite as well: images or scenes with a very oblique light sources should exaggerate depth. Our experiments have tested a very limited stimulus – a depiction of a square and its shadow against a textured background viewed binocularly. There were no other cues to establish its true size or distance in the image nor its relation with the experimental room and its lighting. These factors may have contributed to both the mean value of the relation between shadow offset and depth (a ratio of 1.4) and the large individual differences. Additional experiments with more cues to depth, distance, and lighting are needed to evaluate the generality of our findings.

## Supplementary Material

Supplement 1
