# Supplementary material for: Scaling depth from shadow offset
Source: J Vis. 2021 Nov 29;21(12):15. doi: 10.1167/jov.21.12.15 (PMC8631054; doi:10.1167/jov.21.12.15)
Supplement: Supplement 1 [file jovi-21-12-15_s001.pdf]

Supplementary Material

We consider a simple visual scene consisting of a fronto-parallel square lying a distance of d from the observer and in front of a background surface a distance of D (Fig. S1). We encode the position in depth of the square relative to the background by its relief $r = d/D$. We assume gaze is aligned with the edge of the object, which casts a shadow on the background surface. The tilt of the light source is indicated by the direction of displacement of the shadow, but the slant θ of the light source remains uncertain. We wish to understand the posterior distribution of the relief, conditioned on the observed angular shadow offset ϕ.

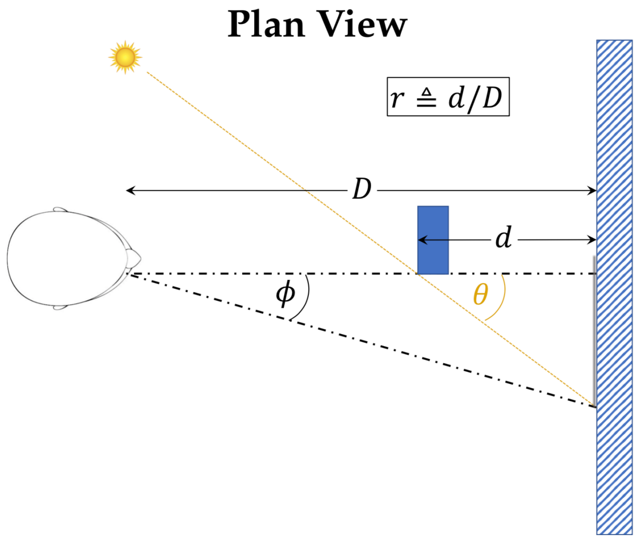

Figure S1. Plan view of geometry of shadow observation

Bayes' rule relates the posterior distribution $p(r|\phi)$ of the relief r, conditioned on the observed angular shadow offset ϕ to the likelihood $p(\phi|r)$ and the prior $p(r)$ over the relief:

$$p(r|\phi) \propto p(\phi|r)p(r) \quad (1)$$

Observe that if the angular shadow offset ϕ is observed accurately, then it must satisfy $D \tan \phi = d \tan \theta$. Thus $\theta = \tan^{-1}\left(\frac{1}{\mathrm{r}} \tan \phi\right)$. Consequently,

$$p(\phi|r) = \left|\frac{d\theta}{d\phi}\right| p(\theta|r) = \frac{r}{r^2 \cos^2 \phi + \sin^2 \phi} p(\theta|r) \quad (2)$$

Note that this expression ignores sensory noise in the observer's estimate of the shadow offset ϕ. Here we assume that this uncertainty is small relative to uncertainty about the direction of the light source.

Assuming that the slant θ of the light source is uniformly distributed over $[0, \pi/2]$ and is independent of the relief r, we have that

$$p\left(\phi|r\right) = \frac{2r}{\pi\left(r^2 \cos^2\phi + \sin^2\phi\right)} \tag{3}$$

Fig. S2 shows the log likelihood $\log p\left(\phi|r\right)$. Note that for tight shadows, the likelihood is strongly peaked near zero relief, whereas for loose shadows, larger relief becomes more probable.

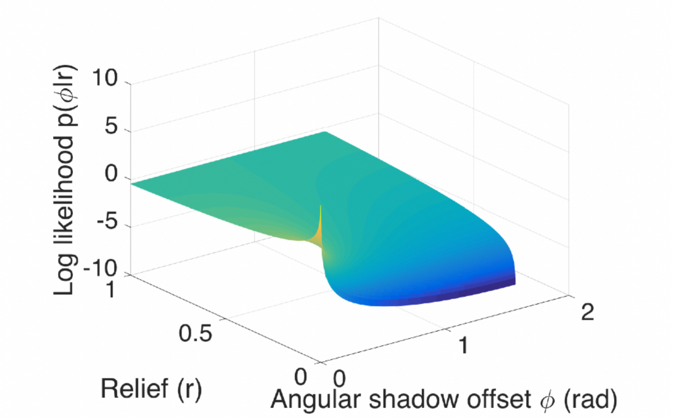

Figure S2. Log likelihood $\log p\left(\phi|r\right)$ of shadow offset ϕ given relief r.

The posterior distribution over relief is also determined by the prior over relief $p(r)$:

$$p\left(r|\phi\right) \propto \frac{2r}{\pi\left(r^2 \cos^2\phi + \sin^2\phi\right)} p(r) \tag{4}$$

Fig. S3 shows this prior estimated empirically from the SYNS dataset (Adams et al., 2016) using the methods of Ehinger et al, 2017. Here we considered only indoor scenes and depth edges near the horizon, although results are similar if we include all elevations. Note that the prior is strongly biased to low relief.

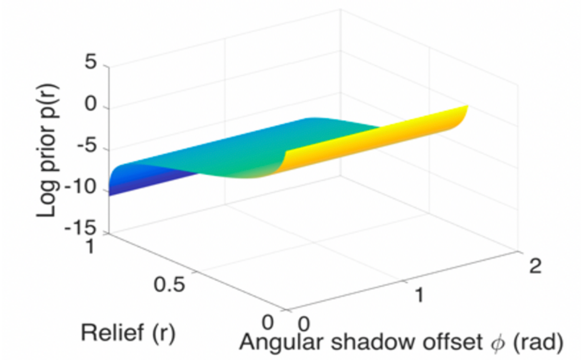

Figure S3. Log prior $p(r)$ *of* relief.

Fig. S4 shows the resulting posterior distribution over relief, and Fig. S5 shows the minimum mean-square error (MMSE) solution, i.e., the expected relief $E[r|\phi]$ given the observed shadow offset ϕ. These results indicate that a Bayesian observer should report low relief for tight shadows and increasing relief as the shadow offset increases. Fig. S6 shows the depth estimates predicted by this Bayesian model in comparison to the GLD model.

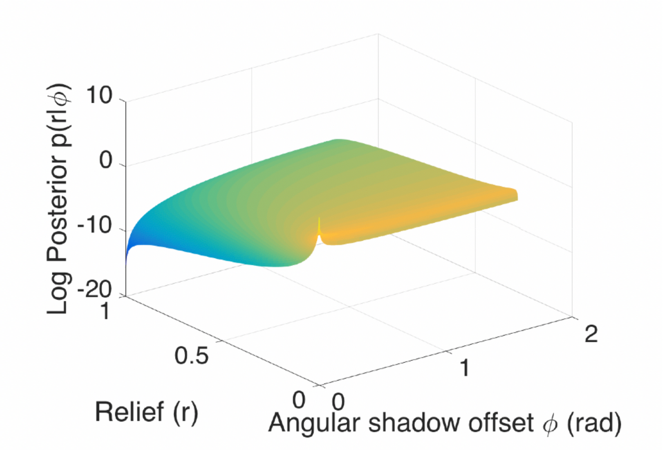

Figure S4. Log posterior p(r|ϕ) of relief r given shadow offset ϕ.

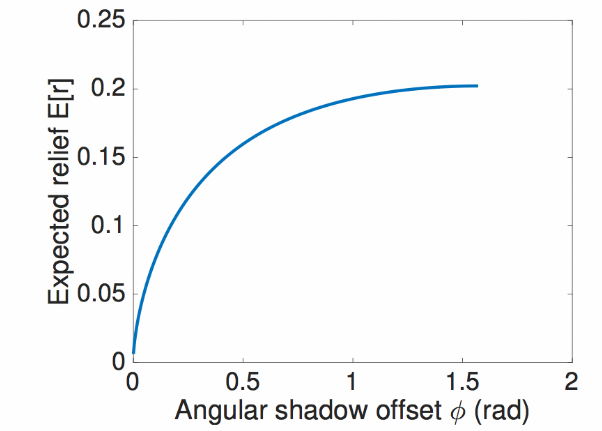

Figure S5. Expected relief $E[r]$ as a function of shadow offset ϕ

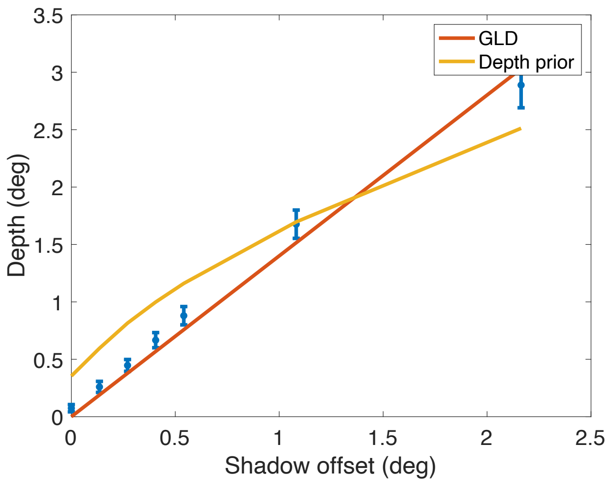

Figure S6. GLD and Bayesian model with empirical depth prior and uniform light prior.

While the Bayesian model fails to match the human data exactly, predicting a compressive scaling rather than the roughly linear scaling observed, it does roughly match the range of human judgements. Note that the bias toward lower relief encoded in the empirical prior is critical for generating estimates in this range. Although the likelihood peaks for small reliefs r, it is far from symmetric: for small r, it is proportional to r, but for large r it is proportional to $1/r$. Thus, while the peak occurs at relatively

small values of r, the distribution has a heavy positive tail. If a flat prior over r is employed, the MMSE model will generate relatively large estimates of r to avoid the heavy penalty that would otherwise be paid if the true relief turns out to be in that tail. The empirical prior prevents this by down-weighting the probability of those large reliefs.

We also considered the possibility of a non-uniform probability distribution over the slant ϕ of the light source. In particular, we modeled the light source slant as a two-parameter beta distribution transformed to [0, 90] degree, and identified the parameters that minimize the weighted squared deviation between the human depth judgements and the expected depth. (Weighting by the precision of the human judgements for each shadow offset corrects for the heterogeneity in the human data apparent in Fig. 6 of the main text). We evaluated a model that combines this light prior with a flat depth prior and a second model that combines the light prior with our empirical depth prior.

Table S1 shows the resulting optimal estimates for the two parameters of the beta distribution, and the mean light source direction for the two models, and Fig. S7 shows the predictions of the resulting Bayesian model with flat and empirical depth priors. This exercise reveals that a broad but non-uniform prior over the light source can account for the human depth judgements just as well as the generic light source model.

Table S1. Estimated parameters of the beta distribution modeling illumination slant.

**Depth prior**	α	β	**Mean (deg)**
Flat	2.54	0.558	73.8
Empirical	4.15	5.85	37.4

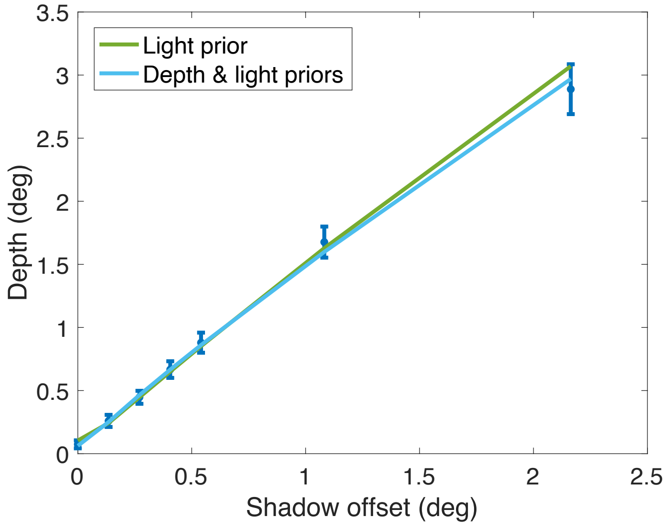

Figure S7. Bayesian model with beta distribution light prior and flat (green) or empirical (blue) depth priors.

To compare model fits to the human depth judgements we measured fit using the squared deviation, weighted by inverse variance, and performed a leave-one-out cross-validation across our 25 observers, which controls for overfitting and differences in model complexity. Fig. S8 shows the resulting RMS errors for the models considered here. We found that the GLD and Bayesian models with both depth and light priors did not differ significantly in their agreement with the human data (p = 0.47), but were both statistically superior to a maximum likelihood (ML) and a Bayesian model with empirical depth prior and flat light prior ($p < .05$).

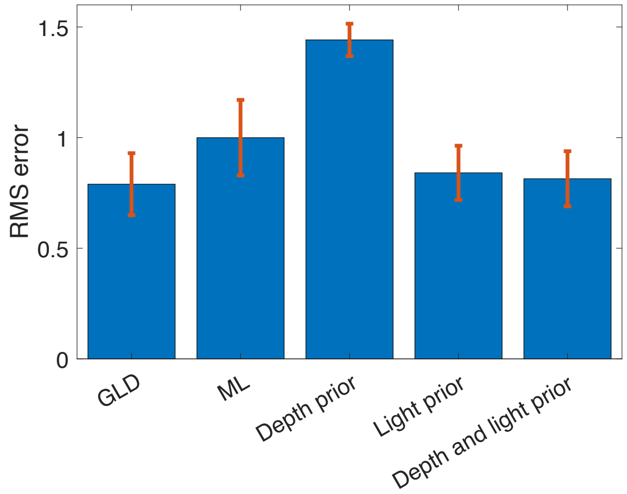

Figure S8. Weighted RMS errors (mean and standard error) of each model against the human data using a leave-one-out cross-validation analysis. **GLD**: Generica light source model. **ML**: Maximum likelihood model. **Depth prior**: Bayesian model with empirical depth prior and flat illumination model. **Light prior**: Bayesian model with flat depth prior and beta distribution model for illumination slant. **Depth and light prior**: Bayesian model with empirical depth prior and beta distribution model for illumination slant.

Note that both the depth and light prior are found to be important. Assuming a flat depth prior and fitting a beta light prior results in a significantly poorer fit (p = 0.0047) to the human data and also an implausible light prior that is heavily biased toward extremely oblique light sources just grazing the surface (slants near 90 degree – Fig. S9). Incorporating the empirical depth prior leads to a more reasonable, roughly symmetric light prior peaking at 37.4 degree slant.

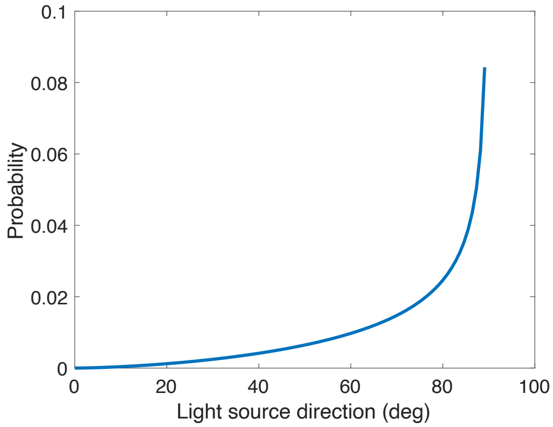

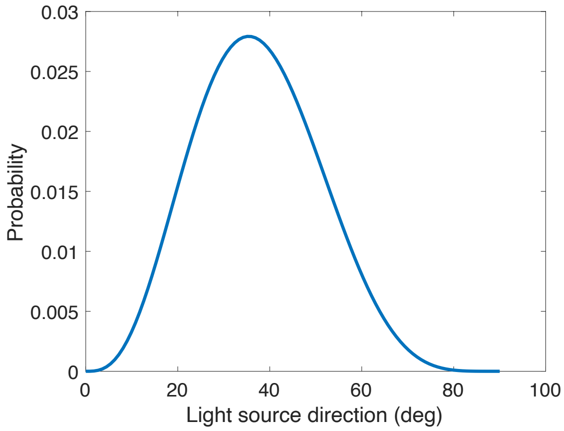

Figure S9. Estimated priors for the illumination slant ϕ when assuming a flat depth prior (left) and when using our empirical depth prior (right).

Such a light prior reflects a bias away from light sources with very small or very large slants. Could there be an empirical basis for such a prior? Note that a small light source slant would entail a light source that was either in front of the observer's eyes, which would make it a visible occluder, or behind the head, which should cast a visible shadow of the head. Absence of these visual cues should therefore bias the observer away from this solution.

What about the bias away from very oblique light sources (large slants)? For the shadows cast below the square one possible explanation could relate to our experience with the sun. If the sun is very oblique to the view vector, its elevation must be close to 45 degree for it to cast a 45 degree shadow, and in Toronto, where the experiments were conducted, the sun reaches this elevation only between the months of March and September. Lower solar elevations are much more common and this should bias the observer away from solutions entailing very oblique light sources.

For indoor lighting, very oblique light sources would have to be in front of the observer to be below the ceiling or above the floor. In either case, the light source would often be visible to the observer were it present. Thus absence of a visible light source should bias the observer away from this solution.
